# Metabolomic Analysis of Pollen Grains with Different Germination Abilities from Two Clones of Chinese Fir (*Cunninghamia lanceolata* (Lamb) Hook)

**DOI:** 10.3390/molecules23123162

**Published:** 2018-11-30

**Authors:** Seif Aldin Dawina Abdallah Fragallah, Pei Wang, Nuo Li, Yu Chen, Sizu Lin

**Affiliations:** 1 College of Forestry, Fujian Agriculture and Forestry University, Fuzhou 350002, China; kingdawina@yahoo.com (S.A.D.A.F.); wp18259199178@163.com (P.W.); linuo1993125@163.com (N.L.); 2 Faculty of Natural Resources and Environmental Studies, University of Kordofan, Elobied 160, Sudan; 3 State Forestry Administration Engineering Research Center of Chinese Fir, Fuzhou 350002, China; 4 Key Laboratory for Forest Adversity Physiological Ecology and Molecular Biology, Fuzhou 350002, China

**Keywords:** Chinese fir, metabolomics, in-vitro pollen germination, metabolic differences

## Abstract

Pollen grains produce certain metabolites, which can improve or inhibit germination and tube growth. Metabolomic analysis of germinating and growing Chinese fir pollen has not been reported. Therefore, this study aimed to analyse metabolites changes, content and expression in the germinating pollen of Chinese fir. To understand the metabolic differences, two clones from Chinese fir were selected. Metabolomics analyses were performed on three stages (1-, 24- and 48-h) during in vitro pollen germination. The metabolites profiles at different time points were analyzed by using liquid chromatography-mass spectrometry. The results showed that 171 peaks were screened; the corresponding differential metabolites of 121 peaks were classified into nine types of substances. The expression of metabolites showed significant differences across and between clones, and the variation was evident at all germination stages. The expression was obvious at the early stage of germination, which differed clearly from that of the late stage after pollen tube growth. Moreover, the metabolites were mainly enriched in 14 metabolic pathways. Pollen germination and tube growth and metabolites expressions changed per incubation time. Since this work is preliminary, we suggest further investigations to understand the relationship between the differential metabolites and pollen development, and factors affecting pollen germination process.

## 1. Introduction

Chinese fir (*Cunninghamia lanceolata* (Lamb.) Hook), is an evergreen conifer tree belonging to the Cupressaceae family. This tree is native to southern China and it is also distributed in northern Vietnam [1]. It is a principal indigenous tree species that occupies approximately 25% of plantations in the subtropical areas of Southern China [1,2]. In addition, it is one of the major commercial conifer species in China, because it is an important tree for excellent timber production [3,4]. Chinese fir is a wind pollinated species, so its pollen and germination ability is greatly affected by external factors and the surrounding environment such as temperature, precipitation, wind power and topography of the area, during its pollination period [5]. The pollination period of Chinese fir is from mid-February to late March, and this period also differs due to geographical distribution. After successful pollination, the process of absorption, transport, and metabolism of nutrients; initiation of germination process; and tube elongation of the pollen are affected by calcium and boron ions of the pollen cells [6]. Understanding the genetic variability of this tree is very important especially for conservation and breeding. Knowledge of pollen germination and pollen tube growth are some of the essential factors in the genetic breeding program [7]. The success of any tree breeding program largely depends on the available genetic variability of the germplasm [2]. Pollen grains are the sexual reproductive units and carriers of male genetic material in higher plants and as such, they play a vital role in the breeding programme. Pollen tube formation is a good and simple criterion for growth and pollen development [8]. Thus, pollen germination and pollen tube growth are important materials for morphological, physiological, biotechnological, ecological, evolutional, biochemical, and molecular biological studies [9]. 

Metabolite measurements have been used for decades to characterize biological systems [10,11,12,13]. Metabolomics is defined as the quantitative measurement of the dynamic metabolic responses of living systems. Metabolomics seeks to identify and quantify the complete set of metabolites in a cell or tissue type without bias [13,14,15]. Research on metabolomics aims at understanding the responses of plants to their own or external environmental conditions by performing a comprehensive qualitative and quantitative analysis of these compounds on a dynamic or static basis [16,17]. Metabolite profiling refers to a set of techniques by which a wide range of metabolites can be detected and quantified from biological extracts. Metabolite profiling using gas or liquid chromatography coupled to mass spectrometry can detect various metabolites such as sugars, amino acids, amines, organic acids, and secondary metabolites [18]. Un-targeted profiling approaches have been used by many scientists to obtain an overview of the metabolic diversity in some plant species such as Arabidopsis [19], tomato [20,21] and pepper [22].

In comparison to the wealth of available transcriptome and proteome data, knowledge on metabolites analysis throughout pollen development is quite limited. Most of the metabolomic metabolic studies on pollen focused on a specific group of compounds, which limits the identification of physiologically important metabolites [21]. For example, a study by Castro and Clément [23] focused on a certain set of metabolites like sugars in the lily, and a study by Sangwan [24] focused on amino acids in *Datura metel*. Despite these examples, a more thorough approach covering the role and importance of primary and secondary metabolites during pollen developmental stages is still missing for any species [21], and similar research on Chinese fir still lags behind. 

Currently, there are a growing number of studies about the pollen of the Chinese fir in the fields of its developmental biology and pollination. Most of these previous studies had focused on morphological characterization of genetic variation, ecological and cytological characteristics [2,4,25,26,27,28,29]. However, metabolomics analysis of the pollen of Chinese fir with different germination abilities during in vitro germination has not been investigated. Therefore, the present study aimed to address this deficit. More specifically, the study focused on metabolites and on changes to metabolic pathways during pollen germination. Indeed, the study provided baseline information, which may serve as a reference for further studies.

## 2. Results

### 2.1. Pollen Germination Status of the Selected Clones

**Figure 1:**
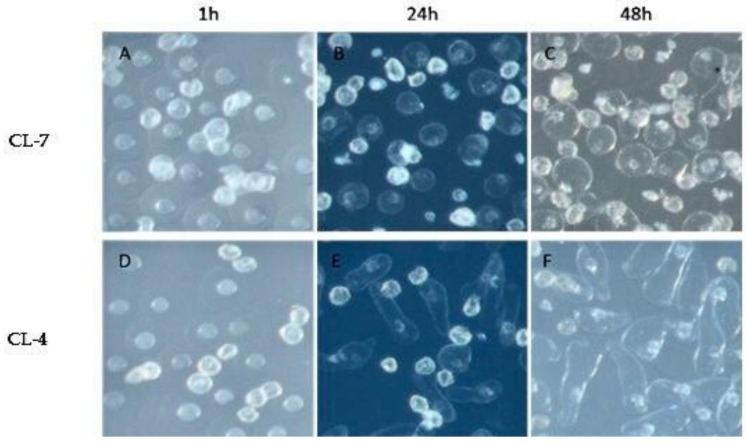
Microscopic observation of pollen germination and tube growth of clone 4 and 7. After one hour of germination, pollen wall rupture was observed in CL-7 (**A**) and CL-4 (**D**); at 24 h, the pollen tube of CL-4 began to germinate (**E**), no pollen tube germination was observed in the pollen grains of CL-7 (**B**). At 48 h, the pollen tube in CL-7 still shows no pollen tube elongation (**C**), while the pollen tube of CL-4 was extended to twice the diameter of its pollen grain (**F**).

Figure 1 and Table 1 show the status of pollen germination and pollen tubes of CL-4 and CL-7 at three developmental stages (1, 24 and 48 h). Germination of the two clones shows variation between them. It is clear that CL-4 has good pollen germination ability and pollen tube growth as compared to CL-7.

### 2.2. Metabolites Variations in the Two Clones (CL-4 and CL-7) in Germinating Pollen

**Figure 2:**
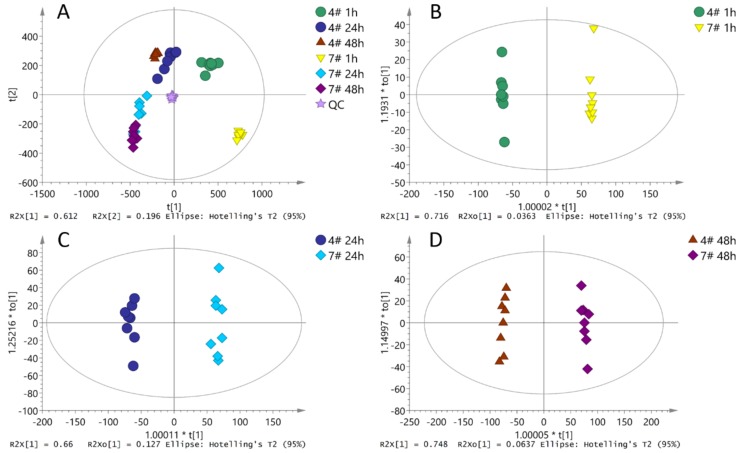
Multivariate statistical analysis, (**A**): principal component analysis (PCA); (**B**): orthogonal partial least squares-discriminant analysis (OPLS-DA) analysis at one hour; (**C**): OPLS-DA analysis at 24 h; (**D**): OPLS-DA analysis at 48 h.

The results of principal component analysis (PCA) of each sample (Figure 2A) showed that the pollen samples at different germination time had obvious degrees of separation. This variation reflects the metabolites changes of the two clones (CL-4 and CL 7) at different times of germination. The results of orthogonal partial least squares-discriminant analysis (OPLS-DA) (Figure 2B–D) indicate that at each time point of pollen germination, the pollen samples of the two clones had a significant spectral separation, indicating that the metabolic differences between pollen of the two clones at different time points were statistically significant.

### 2.3. Differential Metabolites during Pollen Germination

**Figure 3:**
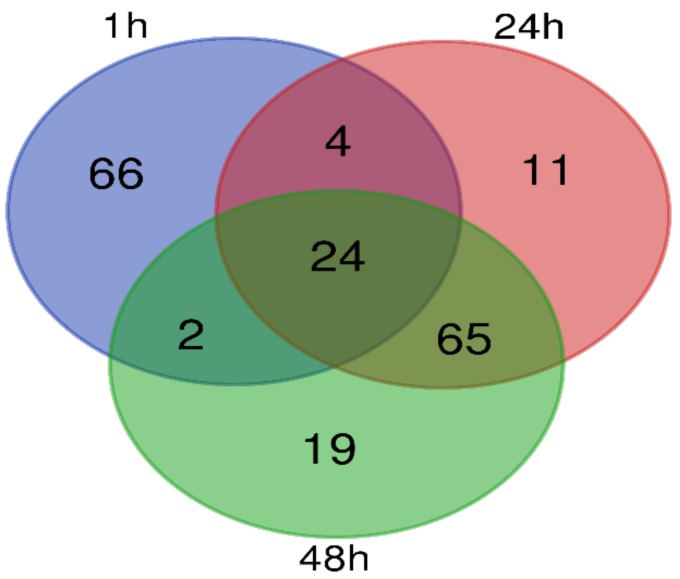
Differential metabolites during pollen germination and tube growth. Venn diagram shows the overlapping and stage-specific differential metabolites from the three stages (1, 24, and 48 h).

Based on the detected peak areas of metabolites, retention time and precise molecular weight in LC-MS, and total particle flow chromatography the chemical structures of the main chromatographic peaks of the pollen samples were identified. 171 peaks were screened, out of which 121 peaks were further classified into nine different class of substances, including vitamins, hormones, fatty acids, organic acids, glycosides, flavonoids, lipids, amino acids and peptides, and amines. Figure 3 shows the number of different metabolites screened throughout the three stages of pollen development as recorded in the control experiment at one hour and others at 24 and 48 h series. Twenty-four of the identified metabolites were common to all developmental stages. The stages of 1 and 24 h shared four metabolites whereas the stages of 1 and 48 h shared two metabolites in common. In addition, 65 metabolites were found to be common to 24 and 48 h stages. However, 66, 11 and 19 metabolites were exclusively specific to 1, 24 and 48 h stages, respectively.

### 2.4. Metabolites Expression and Changes during Pollen Germination

**Figure 4:**
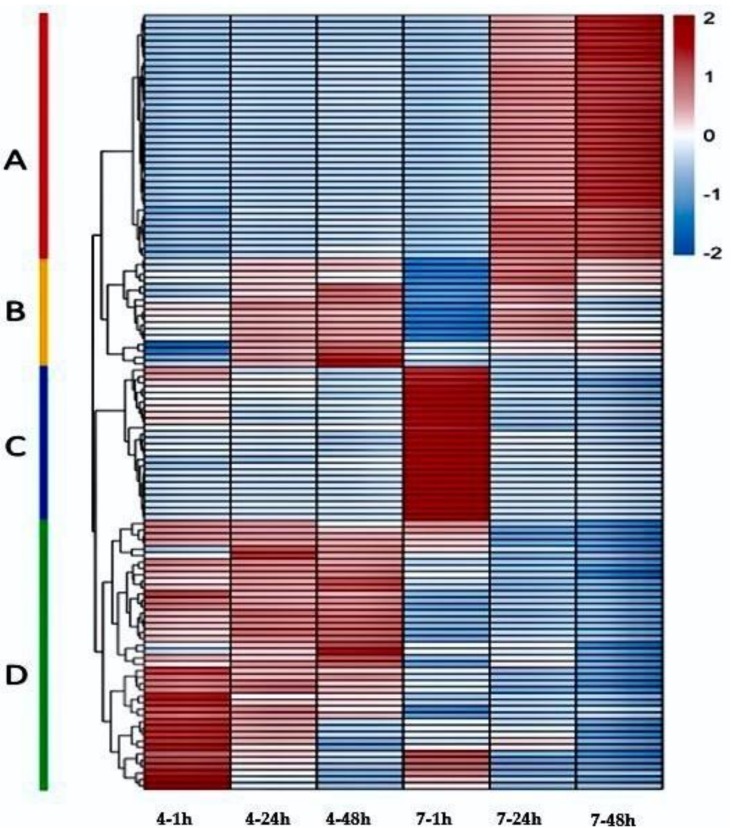
Heatmap showing the differences in the expression levels of metabolites between pollen of the two clones (CL-4 and CL-7) at different germination stages. The sub-maps showed the changes in representative of the differential metabolites at 1, 24, and 48 h of in vitro germination of Chinese fir pollen grain. A color-coded bar on the right represents the value of the metabolite intensity in pollen samples. Colored based on expression and changes of metabolites, red represents high expression whereas blue represents low expression.

The expression of metabolites is shown in the heatmap (Figure 4). The findings indicate high differences in the metabolites of the two clones during pollen germination. Based on the expression status of metabolites of the two clones at different time periods, the metabolites were divided into four categories represented by A, B, C, and D as indicated in Figure 4 and in Supplementary data (Sheet S1). Furthermore, the metabolites in these four categories are grouped into nine types of compounds (Table 2).

The metabolites of category (A) include 38 metabolites. These metabolites are mainly fatty acid, hormones and vitamins compounds (Table 2). The expression levels of these metabolites are low in both clones at early stage of pollen germination. The expression increased after 24 and 48 h of germination. Again, the expression levels of the metabolites are higher in CL-7 than in CL-4. 

The metabolites of category (B) include 17 compounds, most of which are fatty acids (Table 2). The expression levels of these metabolites in the two clones are also very low at the early stage of germination. This expression is slightly higher in CL-4 than in CL-7. Generally, the expression pattern of these metabolites in pollen germination process increases with increase of time in the two clones. 

The metabolites of category (C) include 25 metabolites. These metabolites are mainly flavonoids and glycosides (Table 2). The expression levels of these metabolites in the two clones are significantly different at the early stage of germination, where the expression level in CL-7 was significantly higher than that of CL-4. However, after 24 and 48 h of the pollen germination, the expression levels of the metabolites in the two clones are reduced to lower levels with non-significant differences.

In category (D), 41 metabolites are recorded. These metabolites are mainly fatty acids and lipids compounds (Table 2). These metabolites are highly expressed or up-regulated in both clones at the early stage of pollen germination. The expression levels of these metabolites in the two clones showed significant differences during the whole process of pollen germination. In the CL-4, these metabolites showed a high level of expression at all stages, while in the CL-7 the expression was extremely low, except for a few metabolites, which showed relatively high expression levels at the early stage of pollen germination. At 24 h after germination, the expression level in CL-4 was slightly decreased, but the overall expression was still at a higher level. The expression levels in CL-7 decreased rapidly showing a downward trend, which was significantly different from those in CL-4. At 48 h after germination, the expression of category D metabolites in CL-4 further decreased, showing a down-regulated trend. The expression in CL-7 was similar to that at the stage of 24 h. Therefore, the difference in the expression of metabolites was no longer significant at 48 h in the pollen of CL-7. 

### 2.5. Metabolites Content Changes during Pollen Germination

**Figure 5:**
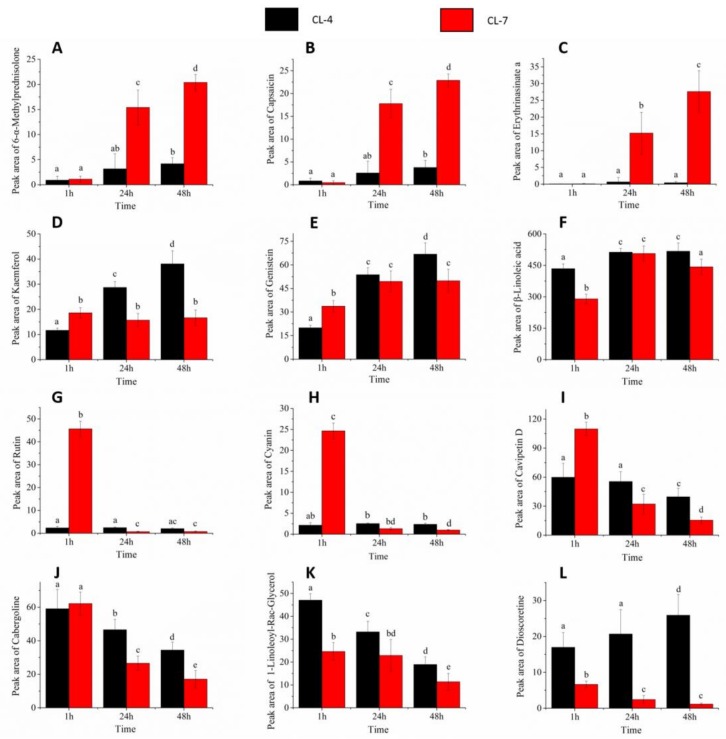
Histograms showing the differences in metabolites content in the pollen of the two clones of Chinese fir at the three germination stages. (**A**): 6-α-methylprednisolone, (**B**): capsaicin, and (**C**): erythrinasinate a are from category (A) metabolites; (**D**): kaempferol, (**E**): genistein, and (**F**): β-linoleic acid are category (B) metabolites; (**G**): rutin, (**H**): cyanin and (**I**): cavipetin D are category (C) metabolites; (**J**): cabergoline, (**K**): 1-linoleoyl-rac-glycerol and (**L**): dioscoretine are category (D) metabolites. The lower case letters on bars represents the levels of significance. Means with the same lower-case letters at top of the bar do not differ significantly at (*p* < 0.05).

Twelve different metabolites were selected randomly from the four categories of metabolites (three metabolites from each category). The content levels of the selected metabolites at different germination intervals are shown in (Figure 5). The metabolites were represented by 6-α-methylprednisolone, capsaicin, and erythrinasinate a, which were selected from category (A) metabolites; kaempferol, genistein and β-linoleic acid selected from category (B) metabolites; rutin, cyanin, and cavipetin d selected from category (C) metabolites; and cabergoline, 1-linoleoyl-rac-glycerol, and dioscoretine selected from category (D) metabolites. These twelve metabolites showed significant differences in the metabolites contents in the pollen of the two clones. Furthermore, the changes in the contents of the selected metabolites (in Figure 5) are similar to the expression levels in the heatmap (Figure 4).

### 2.6. Enrichment of Differential Metabolic Pathways

**Figure 6:**
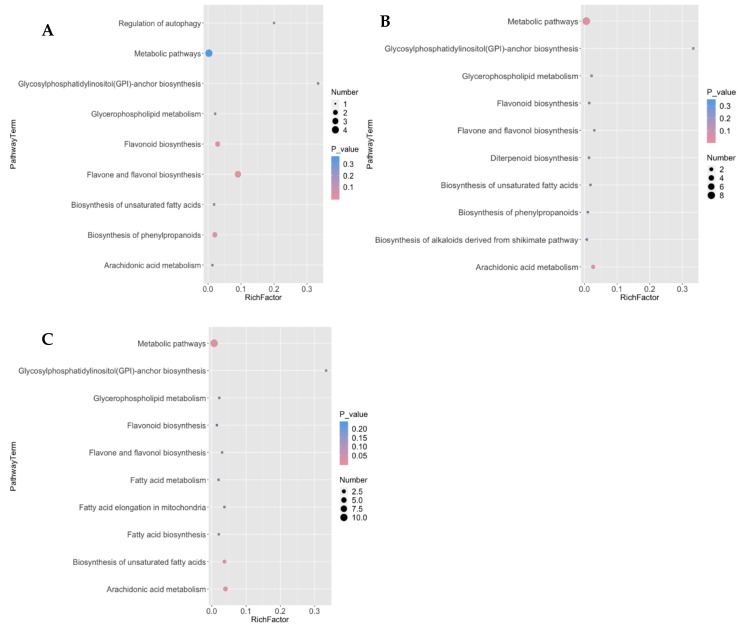
KEGG enrichment of differential metabolic pathways in pollen of the two clones at the three developmental stages (**A**): KEGG enrichment of metabolic pathways at 1 h; (**B**): at 24 h; (**C**): at 48 h. A color-coded bar on the right represents the levels of significance. The pink color indicates extremely significant levels at (*p* < 0.01) while blue color indicates significant levels at (*p* < 0.05). The circles represent the the number of metabolites involved or enriched in the pathway. Enrich factor refers to the ratio of the number of differential metabolites expressed in the corresponding pathway to the total number of metabolites annotated by the pathway. The greater the value the greater is the degree of the enrichment.

The results in Figure 6 show that, the differential metabolites of the two clones during pollen germination are mainly enriched in fourteen metabolic pathways, including flavonoids and flavonol biosynthetic pathways, protein biosynthetic pathway (Glycosylphosphati-dylinositol (GPI)-anchor biosynthesis), arachidonic acid, metabolic pathways, biosynthesis of phenylpropanoids, glycerophospholipid metabolism, regulation of autophagy, diterpenoid biosynthesis, biosynthesis of alkaloids derived from shikimate pathway, fatty acid elongation in mitochondria, fatty acid biosynthesis, fatty acid metabolism and unsaturated fatty acid biosynthesis. At the different intervals of pollen germination, the differential metabolites enrichment of the two clones was different. 

The glycosylphosphati-dylinositol (GPI)-anchor protein showed significant difference in the pollen of the two clones between 1 and 48 h at *p* < 0.05 level of significant. At 24 h, the GPI showed a significant difference (*p* < 0.01) from 1 and 48 h. In addition, there were significant differences in the contents of flavonoids, flavonols in the pollen of the two clones at one hour of incubation. At 24 h, there was a significant difference in the level of arachidonic acid, metabolic pathways, and related metabolites across the clones and time intervals. At 48 h, there were also significant differences between arachidonic acid and the related fatty acids biosynthetic pathways (Figure 6). 

## 3. Discussion

Research on pollen and the application of pollen as breeding materials is very crucial in the field of propagation and development of tree breeding globally. The study of pollen viability and germination activities is one of the basic tasks for breeding programs [30]. It is obvious that the development of Chinese fir sector depends on the processes occurring during germination and growth of the pollen tubes. In this case, knowledge about metabolites changes in germinating and growing pollen tubes is useful in plant breeding because plant organs produce secondary metabolites that have an ability to improve or inhibit germination and pollen tube growth. It is well-known that during the stages of pollen growth and development, the content of organic matter and metabolites will change because germinating pollen accumulates chemical substances [31]. Normally developed pollen contains saccharides, proteins, nucleic acids, and inorganic substances during germination [31,32]. The accumulation of these substances during germination and pollen tube growth stages are important for the development of pollen [32].

In this study, our findings showed that the pollen from the two clones have different abilities of germination (Table 1). The germination activities and the metabolites expression patterns during pollen germination of the two clones are also significantly different. Furthermore, the identified metabolites during the whole germination process changed with changes in the time of incubation. The major reasons for these changes probably are based on the differences in genetic backgrounds of the two clones and metabolites accumulation during pollen germination. Metabolites changes indicate that substances such as hormones, sugars, fatty acids, organic acids and proteins affect pollen germination and pollen tube growth during the germination process [31,32].

Interestingly, metabolites expressions and changes are not uniform across and between the clones at all germination stages. This variation is evident in the pollen of the two clones at the different germination time. For example, the metabolic expression prior to pollen tube germination differed clearly from that during and after pollen tube growth. At the time increases during the pollen germination stages, different physiological activities were also observed.

In the early stage at 1 hour of pollen germination, the main physiological activity was the rupture of the pollen wall (Figure 1). The metabolic differences between the pollen of the two clones at this stage are reflected in category (C) metabolites (Figure 4). Category (C) metabolites are mainly flavonoids and glycosides (Table 2). Generally, flavonoids are considered necessary for pollen germination as well as stimulation of pollen tubes elongation [8,33,34]. Flavonoids are believed to play a key role in the protection of plants against biotic and a biotic stresses [35]. However, in this study it was observed that the expression of flavonoids in the pollen of CL-7 (with the weaker germination activity) was significantly higher than that of pollen of CL-4 (with strong germination activity), indicating that the excessive concentrations of flavonoids may be on the pollen wall. This excessive concentration probably leads to the rupture inhibition in the pollen of the CL-7. Another plausible assumption is that, the pollen of CL-7 contains more oxygen free radicals at the early stages of germination. These radicals are responsible for the inhibition of the physiological activities related to germination, and flavonoids may only have played a defensive role at this stage. These findings are in accordance with the results reported by Zeng et al. [36].

In the middle (24 h) and late stages (48 h) of pollen germination, the main physiological activity was the elongation of the pollen tube (Figure 1). The metabolic differences between the pollens of the two clones are reflected by the expression levels of metabolites in category (A) during this period (Figure 4). Category (A) metabolites includes a variety of organic acids, fatty acids, hormones, and vitamins. These substances may play a role in mediating signal pathways which may subsequently affect cell wall formation, regulate gene expression patterns and induce protein synthesis during pollen germination [37,38]. Plant hormones are one of the most important factors affecting pollen germination [39,40]. In addition to pollen germination, plant hormones can also influence the growth of pollen tubes [41]. In this study, hormonal substances were found in the pollen of both clones, the expression of the hormones between the pollen of the two clones is not significantly different. Based on the findings in this study, we can deduce that, hormones are essential in pollen development, distribution and expression of metabolites in the pollen of the two clones, consistent with the findings reported by Parish et al. [42]. As indicated in Figure 4, the expression levels of category (A) metabolites in the pollen of CL-7 were also significantly higher than those of CL-4, indicating that these metabolites may inhibit the elongation of pollen tube in pollen of CL-7. These results support the findings reported by He et al. [43] which established that fatty acids affect the elongation of pollen tubes.

Lipids, on the other hand, play an important role in pollen development and directing the pollen tube. Lipids are important compounds for stabilization of membranes and for the hydration of pollen upon germination [44]. As shown in Figure 4, the expression levels of category (D) metabolites in the two clones showed significant differences during the whole process of pollen germination. The results in Figure 1 clearly showed that CL-4 has good pollen germination ability and longer pollen tube elongation compared to CL-7. The expression levels of category (D) metabolites in CL-4 were also significantly higher than those of CL-7 during the whole germination. Since most of the metabolites in category (D) are mainly lipids compounds, this probably promoted the expression levels of category (D) metabolites which in turn results in the tube elongation in CL-4, indicating the importance of lipids in pollen germination and pollen tube elongation. These findings are in agreement with Rodriguez-Garcia et al. [45].

Variations in the content of the metabolites show that, these compounds are produced and accumulate differently across and between germinating pollen of the two clones. Based on the results presented in Figure 5, we observed that during the entire development process of the pollen grains many secondary metabolites can be found in different developmental stages. The content of these metabolites were different in different pollen of the two clones. By comparing the differences in metabolite content between the pollens of the two clones, our analysis indicated that with the exception of 6-α-methylprednisolone, capsaicin, erythrinasinate a, rutin, and cyanin, the contents of the metabolites in the pollen of CL-4 was higher than that in pollen of CL-7. Based on this, it can be inferred that the pollen of CL-4 has some metabolites with much higher contents during its development, and these metabolites may participate in the pollen germination and tube elongation. On the other hand, the higher contents of (6-α-methylprednisolone, capsaicin, erythrinasinate a, rutin, and cyanin) metabolites in CL-7 probably contributed to slow germination in this clone. These results got supports from other related research conducted by Palevitch et al. and Chen et al. [46,47]. It shows that an increase in capsaicinoid content corresponds to a decrease in germination activity, while the decrease in capsaicinoid content is always accompanied by a corresponding increase in peroxidase activity [46,47]. Thus, results from this study also showed that there is a negative correlation in the capsaicin content in pollen of the two clones, and capsaicin content in pollen of CL-7 is much higher than that in the pollen of CL-4. This may also lead to a low level of peroxidase in the pollen of CL-7, resulting in pollen germination failure as one of the causes of pollen abortion.

Furthermore, the analysis of metabolites gave a comprehensive overview of the active pathways during pollen germination and pollen tube growth. Glycosylphosphatidyl inositol anchored protein (GPI), is a type of integral protein that is involved in the endoplasmic reticulum and plasma membranes on glycosylphosphatidyl inositol [48,49]. It has been reported that GPI-APs play an important role in plant cell growth, cell division and intercellular communication [49]. Based on the research findings in Figure 6, the content of glycosylphosphati-dylinositol (GPI)-anchor in the pollen of the two clones at the three different developmental stages was significantly different. There is a significant difference between the amount of metabolites expressed in the GPI protein biosynthetic pathway of CL-4 and CL-7. The differences indicate that the metabolites in GPI pathway might have played an important role in pollen germination and tube elongation. The metabolites in this pathway might have led to the abortion of CL-7 pollen. However, lack of corresponding GPI proteins involvement in signal transmission of CL-7 pollen suggests that GPI protein may be normally synthesized during the normal development of the pollen. Therefore, the signal reaction mediated by GPI protein can be smoothly carried out, thereby promoting the interaction between the pollen tube and the megaspore tissue during pollen germination. These results concur with the work done by Gao & Guo; Kinoshita & Fujita [48,49].

## 4. Materials and Methods

### 4.1. Description of the Study Area

This study was conducted by collecting pollen from YouXi State-owned forest farm. This farm is located between latitude 25°50’–26°26’ N and longitude 117°48’–118°39’ E in Fujian Province, China.

### 4.2. Collection of Pollen Materials

Mature pollen grains were collected from third-generation seed orchard of YouXi State Farm. Pollen grains were collected from two clones (CL-4 and CL-7) during pollination season of March 2017. The pollen samples were collected from mature male cones after ripening.

### 4.3. Preliminary Pollen Germination Testing 

In the present study, pollen from two clones of Chinese fir was selected to adopt the current advanced metabolomics research method. The two clones were chosen based on a preliminary identification of pollen germination ability of more than 30 clones (non-published data). The two clones with different germination ability were screened out, and germination was promoted by in vitro pollen culture using a basic medium. Briefly, the basic medium consisted of 5% sucrose, 0.01% boric acid, 0.5% agar, 100 mL distilled water and pH was adjusted to 5.8. The medium components were dissolved in boiling water and poured into petri dishes. Then, pollen was distributed on the surface of the cooling media. Each petri dish received 0.05 g pollen. For the germination test, the petri dishes were inoculated in a growth chamber for 12 h in light and 12 h in dark cycles. The relative humidity and temperature conditions set for inoculation were 70% and 25 °C, respectively. 

### 4.4. Determination of Pollen Developmental Stages

Pollen developmental stages were determined using in vitro pollen germination test using the same basic medium as previously mentioned. Three pollen developmental stages were conducted at 1 h (used as a control) and other two stages at 24 and 48 h. The pollen samples were divided into time points based on time of incubation (i.e., 1, 24, and 48 h samples). Each sample was separately harvested to obtain eight biological replicates. The harvested pollen was washed with phosphate-buffered saline (PBS) solution, and then centrifugation was done at 10,000 rpm for 6 min. The centrifugation was repeated once, and pollen was transferred into 1.5 mL Eppendorf tube. Eight replicates per developmental stage were used and a total of 48 samples were prepared for metabolites extraction. All samples were frozen immediately in liquid nitrogen for 50 min and then stored in refrigerator at −80 °C for further analysis. 

### 4.5. Sample Preparation for Metabolites Extraction

The preparation of samples for metabolites extraction was conducted by taking 30 mg of lyophilized sample and transferring to a 1.5 mL Eppendorf tube. Two small steel balls were added to the tube. Then, 20 μL (concentration of 0.3 mg/mL) of 2-chloro-l-phenylalanine were dissolved in methanol as internal standard. A mixture of 500 µL of methanol and water (in a ratio of 7/3 volume by volume) were added to each sample, which were placed at −80 °C for 2 min. The samples were grinded at 60 Hz for 2 min and ultrasonicated at ambient temperature for 30 min after vortexes and placed at 4 °C for 10 min. Samples were centrifuged at 13,000 rpm, 4 °C for 15 min. The supernatants (200 μL) from each tube were collected using crystal syringes, filtered through 0.22 μm microfilters and transferred to autosampler vials for LC-MS analysis as previously described by Ziegler et al. [50]. The vials were stored at −80 °C until used for LC-MS analysis. Quality control (QC) samples were prepared by mixing aliquots from each of the samples. 

### 4.6. Liquid Chromatography-Mass Spectrometry (LC -MS) Analysis

For each stage, eight biological replicates were independently analyzed. In total, 48 samples were analyzed to reduce analysis bias. An ACQUITY UHPLC system (Waters Corporation, Milford, DE, USA) coupled with an AB SCIEX Triple TOF 5600 System (AB SCIEX, Framingham, MA, USA) was used to analyze the metabolic profiling in both Electrospray ionization (ESI) positive and negative ion modes as previously described by Wahyuni et al. [51]. 

An ACQUITY UPLC BEH C18 column (1.7 μm, 2.1 × 100 mm) was employed in both positive and negative modes. The mobile phase consisted of (A) water (containing 0.1% formic acid, *v*/*v*) and (B) acetonitrile (containing 0.1% formic acid, *v*/*v*) and separation was achieved using binary gradient elution system. The elution gradient is shown in (Table 3). The flow rate was 0.4 mL/min and the column temperature was 45 °C. The injection volume was 5 μL as previously described by Wahyuni et al. [51]. All samples were kept at 4 °C during the analysis.

Data acquisition was performed in full scan mode (*m*/*z* ranges from 70 to 1000) combined with Information Dependent Acquisition (IDA) mode. Parameters of mass spectrometry were as follows: Ion source temperature, 550 °C (+) and 550 °C (−); ion spray voltage, 5500 V (+) and 4500 V (−); curtain gas of 35 PSI; declustering potential, 100 V (+) and −100 V (−); collision energy, 10 eV (+) and −10 eV (−); and interface heater temperature, 550 °C (+) and 600 °C (−). For IDA analysis, the range of *m/z* was set as 50–1000, the collision energy was set at 30 eV to identify selected compounds. The QCs were injected at regular intervals (every 10 samples) throughout the analytical run to provide a set of data from which repeatability was assessed as previously described by Want et al. [52].

### 4.7. Data Processing and Analysis

The raw data were converted to common data format (mzML) files using a conversion software program MS converter. Metabolomics data were acquired using the software XCMS 1.50.1 version, and the variables (*m*/*z*, exact molecular weight) were detected. Finally, a three-dimensional data matrix containing an exact molecular weight, retention time, sample name (observations), and peak intensity (peak area) was produced. The variables presented in at least 50% of either group were extracted. Isotope and internal standard was removed from the data set. 

Then, all ions were normalized to the total peak area of each sample in Excel 2007 (Microsoft, Redmond, WA, USA) to achieve a minimum Relative Standard Deviation (RSD). 1986 metabolite ions were acquired in positive ion mode, and 899 metabolite ions were acquired in negative ion mode. The 82.71% of ions in positive ion mode and 80.09% in negative ion mode exhibited less than 30% of RSD, which displayed good reproducibility of the metabolomics method. The metabolite ions which had RSD% less than 30% were used for the further data processing. The positive and negative data were combined to get a combined data set which was imported into SIMCA software package (version 14.0, Umetrics, Umeå, Sweden).

Principle component analysis (PCA) and (Orthogonal) partial least-squares-discriminant analysis (O) PLS-DA were carried out to visualize the metabolic alterations among experimental groups, after mean centering and unit variance scaling. The PCA was conducted to detect the intrinsic variation in the samples from different stages. The PLS-DA was used to distinguish the differences in metabolic profiles between sample groups. The Hotelling’s T-squared distribution (T^2^) region, shown as an ellipse in score plots of the models, defines the 99.9% confidence interval of the modeled variation as previously described by Li et al. [53].

The variable influence on projection (VIP) ranks the overall contribution of each variable to the OPLS-DA model. The VIP value of metabolites greater than 1 demonstrated that it contributed greatly to the separation of sample groups in the PLS-DA models. Therefore, variables with significant contributions to the separation of samples between groups were selected based on VIP > 3.5 and those variables were considered relevant for group discrimination. Overfitting of the PLS-DA models was checked using a permutation test. Therefore, the default 7-round cross-validation was applied with 1/seventh of the samples being excluded from the mathematical model in each round, in order to guard against over fitting as previously described by Commisso et al. [54].

#### 4.7.1. Identification of Differential Metabolites

The differential metabolites were selected on the basis of the combination of a statistically significant threshold of variable influence on projection values obtained from the OPLS-DA model and *p*-value from a two-tailed Student’s *t*-test on the normalized peak areas. Metabolites with VIP values greater than 3.5 and p values less than 0.0001 were included, respectively. One-step Solution for Identification of Small Molecules in Metabolomics Studies (OSI/SMMS) was used to identify differential metabolites using the metabolomics rapid identification and analysis software system (OSI/SMMS) developed by Dalian Institute of Chemical Physics, Chinese Academy of Sciences and Dalian Dasuo Information Technology Co., Ltd. (Beijing, China). The database used was the standard material database established by the Dalian Institute of Chemical Physics and the Dalian Institute of Chemical Physics and Physics, and the Human Metabolome Database (HMDB, http://www.hmdb.ca/) and METLIN (URL: https: //metlin.scripps.edu/) as previously described by Paupière et al. [55]. Furthermore, the identified metabolites were classified to different compounds using the Human Metabolome Database (HMDB, http://www.hmdb.ca/), METLIN (URL: https: //metlin.scripps.edu/) and (http://www.kegg.jp/kegg/compound/).

#### 4.7.2. Metabolic Pathways Enrichment Analysis

To further interpret the biological significance associated with metabolites changes of Chinese fir pollen. Common pathway analysis was based on Kyoto Encyclopeida of Genes and Genomes KEGG (website: http://www.genome.jp/KEGG/pathway.html) metabolic pathway analysis to link differential metabolites to their metabolic pathways. Through the Metabolites Biological Role (MBRole) pathway analysis function, the KEGG ID of the different metabolites was mapped to the KEGG database and obtained the enrichment results of their metabolic pathways as previously described by Li et al. [53]. The parameters considered in the enrichment analysis were metabolic pathway ID number, metabolic pathway name, the number of metabolites involved in the pathway, the *p*-value of the metabolic pathway, FRD (False Discovery Rate) corrected *p*-value, and −log (*p*-value).

## 5. Conclusions

The results of this study clearly demonstrated that metabolomics analysis is a good too to understand metabolites expression and changes during pollen germination and pollen tube growth of Chinese fir pollen. There were pronounced changes in metabolites during the pollen development process. The pollen germination activities, the metabolites expression patterns, and metabolites changes during the whole germination process of pollen of the two clones are significantly different and the differences are meaningful. The metabolites contents in pollen of the two clones changed dynamically as a function of incubation time. Furthermore, the content of metabolic pathways in the pollen of the two clones at three different developmental stages was also significant. This study is only a preliminary study of metabolomics analysis of Chinese fir pollen during in vitro pollen germination. The identified metabolites may have certain effects on various aspects of pollen germination. Therefore, it would be interesting to conduct in-depth further metabolic studies in near future to understand the relationship between different levels of related metabolites and pollen development, and to understand most of the factors affecting pollen germination process.

## Figures and Tables

**Table 1:** Pollen germination and pollen tube growth of clones 4 and 7.

	Pollen Germination (%)			Pollen Tube Growth (µm)		
Clones	1 h	24 h	48 h	1 h	24 h	48 h
CL-4	82.7	87.02	78.01	0.28	0.42	0.93
CL-7	50.53	51.99	50.53	0.27	0.35	0.51

**Table 2:** Classification of metabolites in pollen of the two clones during pollen germination.

Category	Organic Acids	Fatty Acids	Hormones	Vitamins	Flavonoids	Glycosides	Lipids	Amino Acids & Peptides	Amines
A	7	9	8	7	2	1	2	0	2
B	3	8	0	0	3	0	3	0	0
C	1	2	0	2	10	4	4	1	1
D	5	11	1	1	5	3	10	0	5

**Table 3:** Elution gradient.

Time (min)	A (%)	B (%)
0	95	5
2	80	20
4	75	25
9	40	60
14	0	100
18	0	100
18.1	95	5
19.5	95	5

## Supplementary Materials

The following are available online. Sheet S1: Differential metabolites of pollen from the two clones of Chinese fir during pollen germination process, raw data files of the LC-MS analysis and the annotated metabolites data files.
